# Femtosecond Laser Fabrication of High-Linearity Liquid Metal-Based Flexible Strain Sensor

**DOI:** 10.3390/ma17091979

**Published:** 2024-04-24

**Authors:** Cheng Li, Chengjun Zhang, Haoyu Li, Zexiang Luo, Yuanchen Zhang, Xun Hou, Qing Yang, Feng Chen

**Affiliations:** 1 State Key Laboratory for Manufacturing System Engineering and Shaanxi Key Laboratory of Photonics Technology for Information, School of Electronic Science and Engineering, Xi’an Jiaotong University, Xi’an 710049, China; 13022667987@stu.xjtu.edu.cn (C.L.); haoyuli95@126.com (H.L.); lzx15200839718@stu.xjtu.edu.cn (Z.L.); yuanchen3637@163.com (Y.Z.); houxun@mail.xjtu.edu.cn (X.H.); 2 School of Instrument Science and Technology, Xi’an Jiaotong University, Xi’an 710049, China; zcjun1995@stu.xjtu.edu.cn (C.Z.); yangqing@mail.xjtu.edu.cn (Q.Y.)

**Keywords:** liquid metal, flexible strain sensor, femtosecond laser, wearable monitoring applications

## Abstract

Liquid metal (LM) is widely used in flexible electronic devices due to its excellent metallic conductivity and ductility. However, the fabrication of LM flexible strain sensors with high sensitivity and linearity is still a huge challenge, since the resistance of LM does not change much with strain. Here, a highly sensitive and linear fully flexible strain sensor with a resistive sensing function is proposed. The sensor comprises an Fe-doped liquid metal (Fe-LM) electrode for enhanced performance. The design and manufacturing of flexible strain sensors are based on the technology of controlling surface wettability by femtosecond laser micro/nano-processing. A supermetalphobic microstructure is constructed on a polydimethylsiloxane (PDMS) substrate to achieve the selection adhesion of Fe-LM on the PDMS substrate. The Fe-LM-based flexible strain sensor has high sensitivity and linearity, a gauge factor (GF) up to 1.18 in the strain range of 0–100%, excellent linearity with an R^2^ of 0.9978, a fast response time of 358 ms, and an excellent durability of more than 2400 load cycles. Additionally, the successful monitoring of human body signals demonstrates the potential of our developed flexible strain sensor in wearable monitoring applications.

## 1. Introduction

Flexible electronic devices are bendable and stretchable, compared with electronic devices with traditional rigid silicon-based circuit boards as the core, so they can better fit the human skin surface and better adapt to the complex surface of the human body [1,2,3,4,5]. The application of flexible sensors in a stressful environment has great research value in human–computer interactions [6], medical treatment [7,8], motion recognition [9,10], etc. Among these sensors, strain sensors can sense the movement and deformation signals of limbs by being attached to joints throughout the human body, and can collect human body signal data to help monitor human body conditions. Over the past decade, a large number of flexible wearable strain sensors have been developed, most of which are based on resistive [11,12,13], capacitive [14,15], piezoelectric [16,17], and triboelectric properties [18]. Among all these sensing principles, resistive strain sensors can convert tensile strain into resistance changes and have been widely studied because of their low production cost, simple structure, excellent performance, simple signal reading, and wide application potential. Migliorini et al. reported a resistive strain sensor based on an Ecoflex–gold nanocomposite with a maximum strain of 500% and a gauge factor (GF) of 124 [12]. Kang et al. developed strain sensors by generating cracks in platinum (Pt) film, which demonstrated a GF of over 2000 within a 0–2% strain range [13]. Atalay et al. manufactured a strain sensor through metal deposition and laser grating and achieved an electrode that could maintain its conductivity up to 250% strain [14]. Although these studies have good sensitivity, flexible sensors with high linearity over a wide response range remain a challenge.

In pursuit of flexible strain sensors with ideal sensing performance and structural robustness, the materials used for the electrodes are very important, as they affect the flexibility, stretchability, and stability of the sensor. A variety of materials have been developed to produce high-performance wearable sensors, such as polymer materials [19,20], ionic conductors [18,21], carbon materials [22,23], and metal materials [24,25]. Among the different material types, liquid metal (LM) materials stand out due to their biocompatibility, intrinsically excellent stretchability, and excellent electrical conductivity [26,27,28]. They are widely used in wearable devices [29,30], soft robots [31], flexible actuators [32,33], etc. However, since LM’s strain response to resistance is not that obvious, realizing a highly sensitive and robust flexible strain sensor is still a huge challenge. In addition, although linearity, as an important index to describe the characteristics of the sensor, has a great influence on subsequent data processing, it is still a great challenge to manufacture a flexible strain sensor with high sensitivity and high linearity. Therefore, a new strategy must be invented to obtain highly linear LM-based strain sensors that enable practical applications in physical monitoring.

In addition, the printing quality of LM circuits is one of the key elements that affects sensor performance. To achieve high-quality printing, scientific researchers have made unremitting explorations and developed many unique molding processes such as flat printing methods, including direct writing printing [34,35,36,37], screen printing [38,39], template transfer printing [40,41,42], and microfluidic filling methods, including vacuum perfusion [43,44] and electrowetting [45,46]. Each process has its advantages and still faces many problems and challenges. Among them, the shortcoming of the flat printing process is that it is not selective enough for the substrate. The reason is the lack of understanding of the wetting behavior of the LM on the substrate interface and the inability to solve the problem of poor print quality caused by the insufficient wetting of the liquid metal. Therefore, it is necessary to find a method that can control the surface wettability to improve the accuracy of printed LM circuits.

Herein, we employ femtosecond laser technology to successfully fabricate a highly sensitive and linear fully flexible strain sensor using Fe-doped liquid metal (Fe-LM) as the electrode and polydimethylsiloxane (PDMS) as the substrate. Compared with pure LM, adding Fe into LM to prepare a doped LM can improve the sensitivity while maintaining high conductivity and high ductility of the LM. This is because a mechanical mismatch between brittle electrodes and stretchable polymers causes cracks in the conductive networks, which leads to extremely high resistance. In addition, the sensor can maintain extremely high linearity in a wide strain range. Femtosecond laser technology, a new processing method of cold processing, has the advantage of high peak energy density, small pulse width, high processing accuracy, and small damage threshold to materials. By creating a surface structure based on nanoparticles and giving PDMS a supermetalphobic microstructure, the selective wetting of the liquid metal on the PDMS surface is achieved, forming a new simple, convenient, and high-freedom liquid metal planar printing process. The assembled strain sensor has high sensitivity (GF = 1.18), excellent linearity with an R^2^ of 0.9978, high linearity (0–100% strain), excellent durability (>2400 load cycles), and fast response time (358 ≤ ms). It can be attached to the surface of human skin to achieve sensing and the successful monitoring of various human body signals, such as finger bending, arm bending, knee bending, etc. This preparation strategy demonstrates the great potential for strain sensors in wearable biomonitoring applications.

## 2. Materials and Methods

### 2.1. Materials

The LM used in our experiment was EGaIn (Wochang Metal Co., Ltd., Dongguan, China), which has a melting point of 12 °C. The Fe used was made of high-purity iron powder (Hebei Leber Metal Material Technology Co., Ltd., Xingtai, China) with a specification of 1 micron and a purity of 99.99%. The PDMS layers were finished thin films purchased from Hangzhou Bald Advanced Materials Co., Ltd. (Hangzhou, China).

### 2.2. Preparation of the Strain Sensor

#### 2.2.1. Fabrication of the Substrate

As an extreme manufacturing method, the femtosecond laser is used to construct microstructures on the surface of various materials for the preparation of functional surfaces. Here, we controlled the wettability of a substrate (500 μm in thickness) using a femtosecond laser. The electrode pattern was constructed by a femtosecond laser with typical line-by-line scanning. The femtosecond laser beam (with a pulse duration of 50 fs, central wavelength of 800 nm, and repetition frequency of 1 kHz) from a Ti:sapphire laser system (Coherent, Saxonburg, PA, USA, Librausp 1K-he200) was focused onto the surface of the PDMS sheet with a plano-convex lens (focal length of 200 mm) and a deflection galvanometer in the air. The PDMS substrate was fixed on the stage. The laser power was held constant at 300 mW, and the laser scanning speed was 8 mm/s.

#### 2.2.2. Patterning the Fe-LM Electrode

The PDMS support layers of the Fe-LM electrode were 500 μm thick. Fe-LM was printed directly on the processed substrate using a brush to complete the patterned Fe-LM electrode. Tape with a thickness of 50 μm was glued to the surrounding raw area so that the thickness of the electrode was controlled at 50 μm in the process of printing the electrode.

#### 2.2.3. Assembly of the Strain Sensor

The sensor was prepared by PDMS packaging. First, the ribbon cable was stuck to the Fe-LM electrode of the PDMS support layer for subsequent testing. Then, a cured encapsulation of PDMS was used to form the final sensor (Figure S1, Supplementary File). The prepared flexible strain sensor could withstand complex elastic deformation, such as stretching, twisting, and squeezing.

### 2.3. Characterization

The surface microstructures of the samples were observed with a Flex 1000 scanning electron microscope (SEM; Hitachi, Tokyo, Japan). The wettability of the liquid Fe-LM droplets on the sample surface was investigated with a JC2000D contact angle (CA) system (Powereach, Shanghai, China). The resistance was measured with a WK4100 LCR meter (Wayne Kerr Electronics, Bognor Regis, UK) (Figure S2, Supplementary File). All resistance signals were tested at a frequency of 20 Hz. 

## 3. Results

### 3.1. Construction of Resistive Strain Sensor Based on Femtosecond Laser

**Figure 1:**
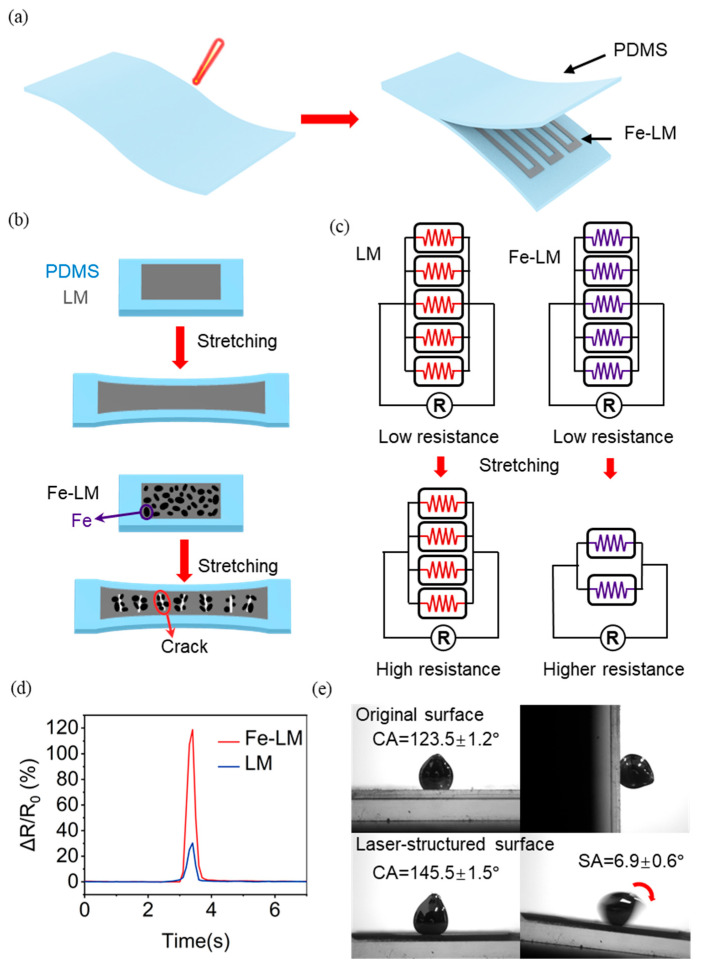
Design and principle of the flexible strain sensor: (**a**) construction of the electrode pattern on PDMS by a femtosecond laser and assembly of the resistance sensor; (**b**,**c**) schematic diagram of the stretching effect of LM and Fe-LM electrodes; (**d**) comparison of signal intensity of different electrodes; (**e**) wettability of the Fe-LM on the laser-structured substrates.

The structural schematic diagram of our proposed resistive strain sensor is shown in Figure 1a, where PDMS is used as the substrate and encapsulation layer and Fe-LM is used as the electrode. The fabrication process (Figure S1, Supplementary File) of the strain sensor began with the preparation of the electrode pattern on PDMS by a selective wetting method using a femtosecond laser. The laser power was 300 mW and the scanning speed was 8 mm/s. The processed PDMS was then cleaned with alcohol and dried. Fe-LM electrodes were printed by a brush onto the PDMS substrate, and ribbon cables were connected to the electrodes for subsequent testing. Finally, PDMS was maintained at 80 °C for 2 h to achieve curing and encapsulation, and the strain sensor was prepared.

The rationale for selecting the Fe-LM electrode over the LM electrode lies in the exceptional ductility and elongation at break exhibited by LM, which results in minimal changes to the conductor’s cross-sectional area during stretching. Consequently, this leads to reduced sensitivity and inadequate resistance response to small strains (Figure 1b). In contrast, the mechanical mismatch between the brittle electrode and the LM caused by doping Fe can cause the conductive network to break down during stretching, resulting in cracks and thus extremely high resistance (Figure 1b). Figure 1c explains the reason for this change. In this simplified model, the LM electrode is considered as a parallel connection of multiple resistors, and the reduction in the cross-sectional area during stretching corresponds to the reduction in the number of parallel resistors. The small change in the cross-sectional area of the LM during stretching corresponds to a small change in the number of parallel resistors, and therefore the change in resistance is small. The Fe-LM electrode, on the other hand, produces cracks locally during stretching, which is regarded as a large reduction in the number of parallel resistors, increasing resistance. It can be seen that Fe-LM-based sensors can significantly improve the sensing performance compared to LM-based sensors (Figure 1d).

The printing of Fe-LM is crucial for the preparation of resistive circuits for sensors. Due to its highly adhesive oxide layer, Fe-LM easily adheres to most surfaces. When Fe-LM is used as a flexible electrode, an LM printing process that can prepare high-resolution patterns by selective wetting and is stable and fast is required. Our femtosecond laser microstructuring technique overcomes the adhesion problem between Fe-LM and the substrate. The Fe-LM droplets exhibit high adhesion on the pristine PDMS surface with a contact angle (CA) of 123.5 ± 1.2° and a sliding angle (SA) > 90°. In contrast, the larger CA of 145.5 ± 1.5° and SA of 6.9 ± 0.6° for Fe-LM was measured on the processed PDMS surface (Figure 1e). This nonstick substrate was defined as a superhydrophobic metallic surface. The nonstick substrate was attributed to the presence of a large number of nanoparticles, which led to a significant reduction in the contact area between the Fe-LM and the surface of the machined area. Although the existence of oxide film makes the contact angle and sliding angle difficult to measure, and there is unreliability [47,48], the adhesion of Fe-LM on different substrates is found to be different through comparison. These results indicate that the femtosecond laser-based fabrication technique could potentially serve as an ideal alternative to template printing, etching, and photolithography with a higher degree of flexibility in the choice of designs based on applicational demands.

### 3.2. Preparation of Fe-LM Electrode

**Figure 2:**
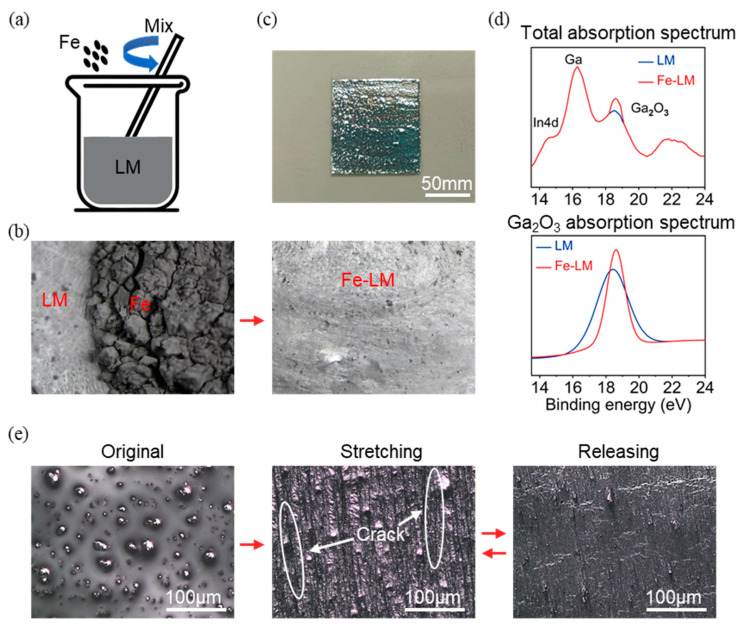
Preparation of Fe-LM and principle of enhancement of signal intensity: (**a**,**b**) preparation process of Fe-LM; (**c**) 1 × 1 cm area of Fe-LM on PDMS; (**d**) XPS spectra of LM and Fe-LM; (**e**) microscope diagram of the stretching effect of Fe-LM electrode.

The fabrication process of Fe-LM is shown in Figure 2a. Iron particles with a size of 1 μm were mixed with LM (1:4 fixed weight ratio) (Figure S3, Supplementary File). Although the density of iron is greater than that of LM, due to the high surface tension of LM, there is a delamination of iron particles and LM, and the iron particles float in pieces above the LM (Figure 2b). After stirring and mixing at room temperature (25.6 °C) for 30 min, it can be observed that the iron particles and LM are completely mixed (Figure 2b).

The most significant feature of Fe-LM is the change in liquid viscosity: the liquid fluidity decreases, and the solid interface adhesion becomes higher. It can be easily coated on PDMS, as shown in Figure 2c, which is Fe-LM in a 1 × 1 cm area. The reason for this phenomenon is that when LM is mixed and stirred with iron powder, the presence of iron particles acts as an abrasive agent, causing the originally dense oxide layer of the liquid metal to break down and enter the interior of the LM, and a new oxide layer reappears on the surface of the liquid metal, which greatly increases the content of the solid components in the liquid. The elements in the Fe-LM were more accurately characterized using an X-ray diffractometer (XPS) (Figure 2d). The absorption peaks of elemental oxygen were located between 14 eV and 23 eV. After peak splitting of the XPS spectra, characteristic peaks of In4d, Ga, and Ga_2_O_3_ could be observed, with an increase in the peak intensity of Ga_2_O_3_ at about 18.9 eV compared to the pre-stirring doping period, proving that additional LM oxides had been added during the doping process.

Due to the addition of Ga_2_O_3_, which has poor tensile properties, Fe-LM produces tiny cracks under a tensile state that would not be produced by LM (Figure 2e). By observing the original Fe-LM, as well as the relaxed and stretched Fe-LM under a microscope, it can be seen that the surface of the original printed Fe-LM is relatively smooth, while during stretching, cracks are generated in Fe-LM perpendicular to the stretching direction. The cracks close after tensile recovery but do not return to their original smooth state (Figure 2e). This type of fracture only occurs in the tensile state but can be generated repeatedly in repeated “loading–unloading” cycles. These cracks cause a reduction in the effective cross-sectional area of the conductor of Fe-LM in the stretched state, resulting in a localized sudden change in resistance increase, which increases the overall sensitivity of the liquid metal resistance to tensile strain.

### 3.3. Wettability Control by Femtosecond Laser

**Figure 3:**
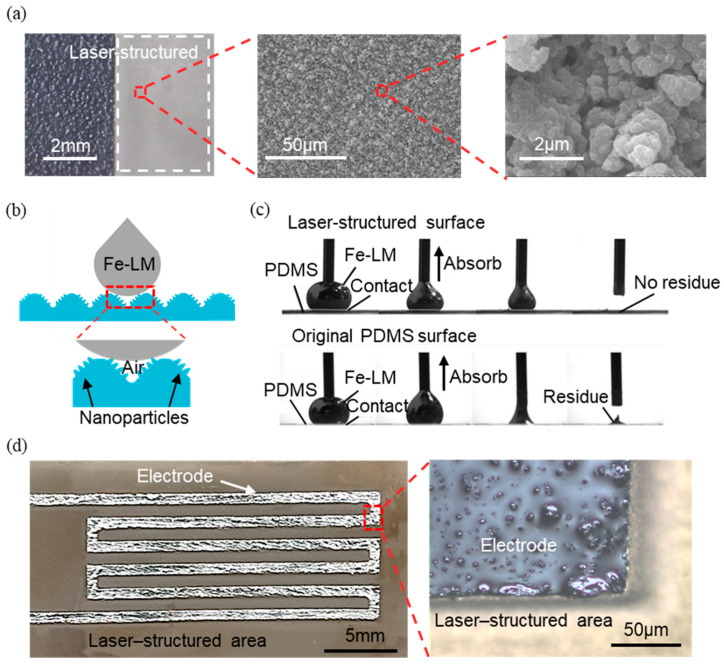
Characterizations and principles of femtosecond laser-structured substrate for printing Fe-LM electrode: (**a**) photo and SEM images of laser-structured substrates; (**b**) schematic of Fe-LM on laser-structured substrates; (**c**) adhesion of Fe-LM on laser-structured substrates and original PDMS surface; (**d**) high-precision printed Fe-LM electrode.

With its micro/nano-processing capabilities, femtosecond laser technology can provide a promising avenue for modulating substrate surface wettability. Figure 3a shows the laser-structured region on a PDMS substrate. The wettability can be modulated very easily on the PDMS film using femtosecond laser technology. It can be seen from the figure that abundant nanoparticles were induced during laser processing. The presence of these abundant nanoparticles led to an air layer and significantly reduced the contact area between the Fe-LM and the substrate surface, achieving the supermetalphobicity toward the Fe-LM (Figure 3b).

The basic principle of Fe-LM printing assisted by the wettability modification of the material surface with the help of a femtosecond laser is well defined, but experimental validation is needed for the reliability of the process. The Fe-LM droplets were extruded with a syringe and sucked up after being pressed onto the PDMS surface, and this behavior was used as an analogy for the Fe-LM printing process (Figure 3c). When Fe-LM is absorbed on the laser-scanned PDMS surface, no residue is left after the absorption is completed. This is because Fe-LM has extremely low viscosity on the rough surface after femtosecond laser treatment, and Fe-LM can be easily taken away from the surface without leaving any residue. When being absorbed on an untreated PDMS surface, Fe-LM sticks to the material, leaving more residue after the absorption is completed. This is because the viscous force of the oxide film wrapped on the outer layer of the Fe-LM to the intrinsic PDMS is greater than its tensile strength limit, which causes the oxide film to break during the absorption process, and the Fe-LM remains on the surface of the material. The extremely different properties of Fe-LM on these two surfaces demonstrate the excellent reliability of the flat printing process.

The 80 μm line width wire printed by the femtosecond laser’s selective wetting method was observed under an optical microscope to show that the Fe-LM was tightly adsorbed at the runner edge and did not remain in the processed area (Figure 3d). The Fe-LM wire had a clear boundary profile and the conductive pattern was generally of high resolution. These findings demonstrate that the utilization of femtosecond laser technology for modulating substrate wetting properties offers a stable and efficient approach for selective wetting in liquid metal printing processes, thereby facilitating the fabrication of high-resolution patterns.

### 3.4. Sensing Properties of the Strain Sensor

**Figure 4:**
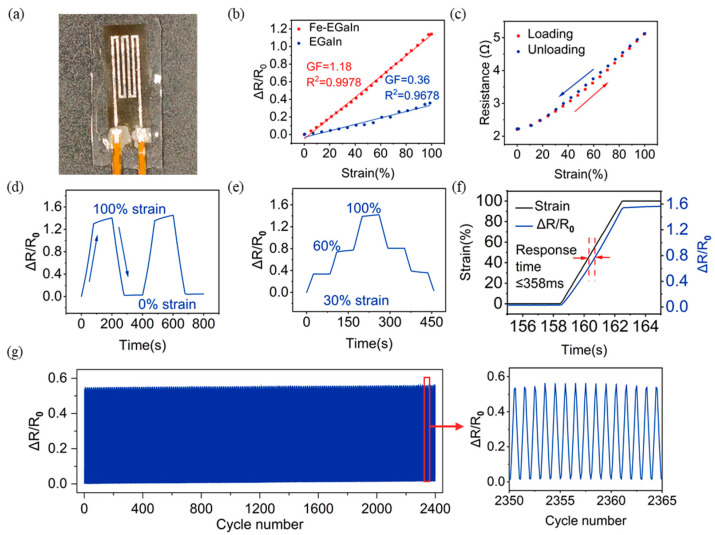
Characterizations of flexible strain sensor: (**a**) photo of flexible strain sensor; (**b**) sensor sensitivity curves with two different types of electrodes; (**c**) resistance change in sensor during the loading and unloading process; (**d**) normalized resistance changes under the same strain for a period of time; (**e**) normalized resistance changes under different strains; (**f**) sensor response time test curve; (**g**) cycling stability at a strain of 50% for 2400 cycles.

The strain sensor we designed consists of interdigital electrodes with a line width of 0.8 mm and a length of 20 mm (Figure 4a). This Fe-LM strain sensor has a variety of superior properties, including high sensitivity, high linearity, low hysteresis error, good stability, and so on. These properties make it a promising candidate for a new generation of flexible strain sensors. In this study, we systematically investigate the sensing performance of Fe-LM strain sensors.

Figure 4 shows the performance of our femtosecond laser-fabricated Fe-LM strain sensor. The sensitivity factor (GF), defined as the slope of the ΔR/R_0_ strain curve, is the basic performance index of the strain sensor. At 0–100% strain, the LM strain sensor has a GF of 0.36 and an R^2^ of 0.9678. The resistance change between 0% and 20% strain is less than 5%, which indicates that the sensor is insensitive to the resistance response to small strains and has poor sensitivity (Figure 4b). In contrast, the Fe-LM strain sensor has a GF of 1.18, which is 328% higher than that before the doping modification, and an R^2^ of 0.9978, with excellent linearity and significantly (Figure 4b). It improves the sensor resolution. This is because the LM, after the doping modification, increases the influence on the conductor cross-section during the stretching process, which makes it easier to change the resistance value and to sense the tensile strain signal.

During the operation of elastic sensor devices, some devices have a null range caused by elastic hysteresis due to poor elasticity, which affects the ability to obtain correct measurement results. The flexible sensor device in this experiment has high elastic strength and elastic recovery speed due to the use of highly elastic materials such as PDMS, and also Fe-LM can flow well within the encapsulation layer, so the device has the advantage of being able to perform forward and reverse travel sensing measurements with a hysteresis error of only 0.04 (Figure 4c).

Stability is a parameter that measures the ability of a sensor device to maintain a stable output. To characterize the stability of the sensor, we tested ΔR/R_0_ held at the same strain for a period of time (Figure 4d) and ΔR/R_0_ held at different strains for a period of time (Figure 4e). The normalized resistance of the sensor drifted by about 10% under 100% tensile strain, but after unloading and relaxing, the normalized resistance could return to the initial value normally and the waveform signals output from the sensor after reloading were highly repeatable. The output curve was basically symmetrical and the step shape was clear after loading to 100% strain in stages and unloading to the relaxation state in stages, and the device still had good output stability.

The sensor, whose response time is a measure of how quickly it responds to the input signal, also exhibited a fast response time of 358 ms during loading and unloading at 100% strain (Figure 4f). The durability of the sensor under long-term cyclic loading is a crucial factor that influences the robust application of the tactile sensor. As shown in Figure 4g, the resistance response of the strain sensor also remained constant after 2400 stretch/release cycles at 50% strain, demonstrating the high durability of the sensor. These excellent properties demonstrate the potential of Fe-LM strain sensors for human wearable electronics applications.

### 3.5. Applications of the Strain Sensor in Human Body Signal Monitoring

**Figure 5:**
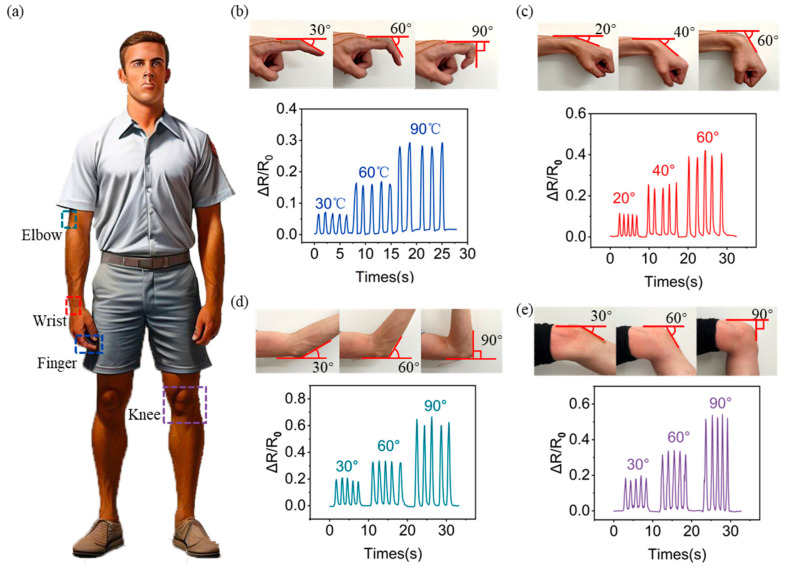
Wearable strain sensor for mechanical signal monitors: (**a**) different parts of the human body where signals are monitored; real-time monitoring of (**b**) finger bending, (**c**) wrist bending, (**d**) elbow bending, and (**e**) knee bending.

To demonstrate the potential of the prepared Fe-LM strain sensors for wearable monitoring applications, the sensors were attached to different parts of the human body for monitoring (Figure 5a and Figure S4, Supplementary File). All resistance signals were tested using a WK4100 LCR meter at a frequency of 20 Hz.

When the sensor was installed at the finger joint when the finger was bent at 30°, 60°, and 90° in sequence, the change in normalized resistance increased accordingly (Figure 5b). Figure 5c shows the ΔR/R_0_ of the sensor in response to wrist bending. When the wrist was bent at 20°, 40°, and 60° in sequence, the change in normalized resistance increased accordingly. Likewise, when the sensor was attached to the elbow, the sensor could clearly sense different bending amplitudes of the elbow, and the electrical signal was highly repeatable and stable (Figure 5d). Also, Figure 5e shows the ΔR/R_0_ of the sensor in response to knee bending. When the knee was bent at 30°, 60°, and 90°, the change in normalized resistance also increased accordingly. The above results show that the sensor can respond to joint movements (bending angle changes) and the signal value has good stability.

In summary, the experimental results show that the sensor can effectively monitor various mechanical signals (from weak to strong signals) of the human body as a wearable device and can distinguish mechanical signals in different states (strong or weak), demonstrating promising application potential in the field of human–machine interaction and human health monitoring.

## 4. Discussion

We demonstrate a highly sensitive and linear strain sensor with a resistive sensing function, using Fe-LM as the electrode and PDMS as the substrate. Compared with traditional LM, the Fe-LM electrode has improved strain sensitivity due to its cracks generated during the stretching process. Femtosecond laser micro/nano-processing technology can control the wettability of the surface, thereby achieving the rapid and high-precision printing of Fe-LM electrodes. The proposed femtosecond laser can enable programmable, high-efficiency, low-cost, and large-scale electrode pattern fabrication in a simple manner, which is difficult to achieve using traditional techniques such as templates, etching, and photolithography.

The resulting strain sensor exhibits a GF of 1.18, a high linearity from 0 to 100% strain, a fast response time of 358 ms, and a cycle durability of over 2400 cycles. Compared with the reported results of GF, strain range, and cycle number (Table 1), it can be seen that some sensors have an excellent GF or a wide strain range, but it is difficult for them to maintain complete linearity in a wide strain range. Compared with them, the sensor we prepared can maintain good linearity in a wide strain range. At the same time, the cycle numbers are also relatively large. All of these qualities are very helpful in practical applications. Strain sensors were successfully used to monitor various human body signals, demonstrating their application potential in wearable biomonitoring, human–machine interfaces, and soft robotic systems.

## Figures and Tables

**Table 1:** Comparison of the performance of the strain sensor with the reported literature in terms of GF, strain range, and cycle number results.

Materials	GF	Strain	Cycle	Source
Fe-LM	1.18	100%	2400	This work
PAM@CNC/TA-Ag NC hydrogels	1.02	100%	500	[49]
Silver nanoparticle film	1.2 (5%), 2.05 (20%)	20%	1000	[50]
BPTP composite conductive hydrogels	1.4 (0–60%), 6 (60–120%)	120%	1000	[51]
Ultrathin gold nanowires	6.9–9.9	350%	5000	[52]
Pt nanoparticles	7998 (4.17%), 2.6 × 10^8^ (7.2%)	7.2%	1000	[53]
Ecoflex-CB	1.62–3.37	500%	10,000	[54]

## Data Availability

Data are contained within the article and Supplementary Materials.

## Supplementary Materials

The following supporting information can be downloaded at: https://www.mdpi.com/article/10.3390/ma17091979/s1, Figure S1: Preparation of the strain sensor; Figure S2: A WK4100 LCR meter; Figure S3: Resistance change of different weight ratios of Fe-LM; Figure S4: The strain of the sensor at different positions as a function of angle.
