# Supplementary material for: Femtosecond Laser Fabrication of High-Linearity Liquid Metal-Based Flexible Strain Sensor
Source: Materials (Basel). 2024 Apr 24;17(9):1979. doi: 10.3390/ma17091979 (PMC11084944; doi:10.3390/ma17091979)
Supplement: Supplementary file 1 [file materials-17-01979-s001.zip › materials-2949146-supplementary.pdf]

Supplementary Materials for

Femtosecond Laser Fabrication of High-Linearity Liquid Metal-Based Flexible Strain Sensor

Cheng Li[1], Chengjun Zhang[2], Haoyu Li[1], Zexiang Luo[1], Yuanchen Zhang[1], Xun Hou[1], Qing Yang[2] and Feng Chen[1,*]

[1]*State Key Laboratory for Manufacturing System Engineering and Shaanxi Key Laboratory of Photonics Technology for Information, School of Electronic Science and Engineering, Xi'an Jiaotong University, Xi'an 710049, China*

[2]*School of Instrument Science and Technology, Xi'an Jiaotong University, Xi'an 710049, China*

*Correspondence: chenfeng@mail.xjtu.edu.cn

This PDF file includes:

Figures S1 to S4

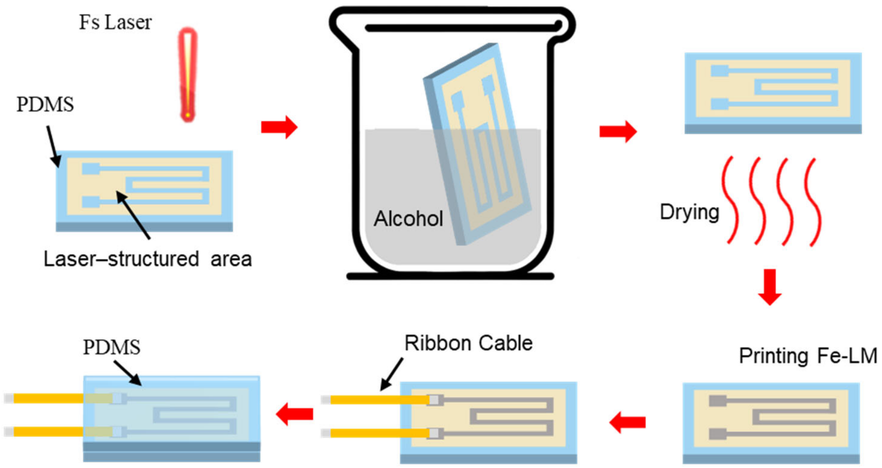

Figure S1. Preparation of the strain sensor.

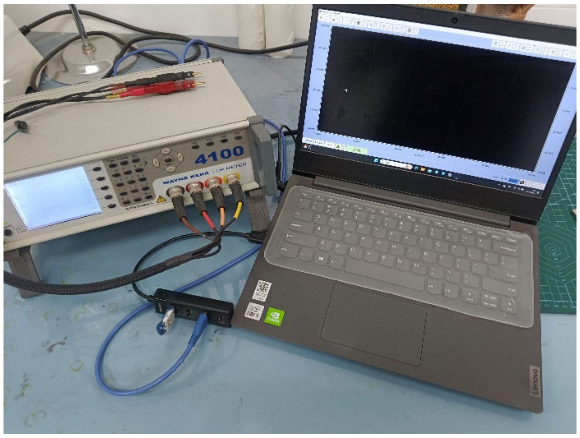

Figure S2. A WK4100 LCR meter.

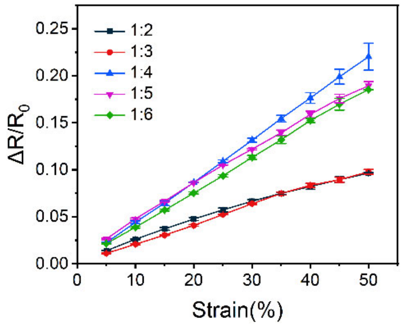

Figure S3. Resistance change of different weight ratios of Fe-LM.

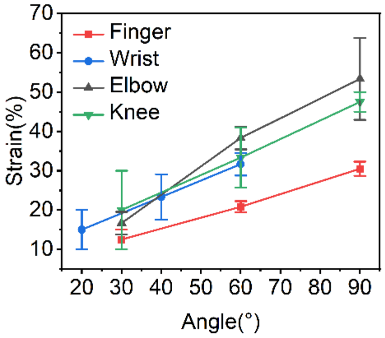

Figure S4. The strain of the sensor at different positions as a function of angle.
